# Correlation of Breed, Growth Performance, and Rumen Microbiota in Two Rustic Cattle Breeds Reared Under Different Conditions

**DOI:** 10.3389/fmicb.2021.652031

**Published:** 2021-04-29

**Authors:** Matteo Daghio, Francesca Ciucci, Arianna Buccioni, Alice Cappucci, Laura Casarosa, Andrea Serra, Giuseppe Conte, Carlo Viti, Breanne M. McAmmond, Jonathan D. Van Hamme, Marcello Mele

**Affiliations:** ^1^Dipartimento di Scienze e Tecnologie Agrarie Alimentari Ambientali e Forestali, University of Florence, Florence, Italy; ^2^Dipartimento di Scienze Agrarie, Alimentari e Agro-ambientali, University of Pisa, Pisa, Italy; ^3^Centro di Ricerche Agro-ambientali “E. Avanzi”, University of Pisa, Pisa, Italy; ^4^Department of Biological Sciences, Thompson Rivers University, Kamloops, BC, Canada

**Keywords:** growth performance, high-throughput sequencing, microbiota, rumen, rustic cattle

## Abstract

The use of rustic cattle is desirable to face challenges brought on by climate change. Maremmana (MA) and Aubrac (AU) are rustic cattle breeds that can be successfully used for sustainable production. In this study, correlations between two rearing systems (feedlot and grazing) and the rumen microbiota, the lipid composition of rumen liquor (RL), and the growth performance of MA and AU steers were investigated. Bacterial community composition was characterized by high-throughput sequencing of 16S rRNA gene amplicons, and the RL lipid composition was determined by measuring fatty acid (FA) and the dimethyl acetal profiles. The main factor influencing bacterial community composition was the cattle breed. Some bacterial groups were positively correlated to average daily weight gain for the two breeds (i.e., Rikenellaceae RC9 gut group, *Fibrobacter* and *Succiniclasticum* in the rumen of MA steers, and Succinivibrionaceae UCG-002 in the rumen of AU steers); despite this, animal performance appeared to be influenced by short chain FAs production pathways and by the presence of H_2_ sinks that divert the H_2_ to processes alternative to the methanogenesis.

## Introduction

Maremmana (MA) and Aubrac (AU) cattle are rustic breeds well suited for diverse farming conditions, due to their ability to efficiently use low-quality dietary fiber (Renand et al., 2002; Sargentini, 2011; Gallo et al., 2014; Bongiorni et al., 2016; Conte et al., 2019). AU is a breed that originated on the Massif Central in southern France and currently is mainly raised for beef production. The MA breed is typically raised in the Maremma region in Central Italy. It was formerly selected as a work animal, but is now raised only for beef production.

It has been suggested that the use of more resilient breeds (rustic breeds) for beef production is desirable to face challenges brought on by climate change (Horizon, 2020). Rustic breeds may better adapt to challenging and diverse livestock systems due to specific genetic traits. Furthermore, the rumen microbiota plays an important role in the adaptive response of animals to environmental challenges (Zhong et al., 2019). Several authors have hypothesized that host genetics can be fundamental in selecting rumen microorganisms and suggested that breeding strategies could exploit the abundance of rumen microbial genes to select animals with desirable traits (Roehe et al., 2016).

Molecular analysis of rumen liquor (RL) can provide detailed information on the composition of rumen microbiota, and associations between rumen microbial community structure and feeding regimens, diet composition, and host genetics have been made (Li et al., 2019b; Vasta et al., 2019). There is increasing interest in understanding associations between rumen microbiota, rumen metabolism, and animal performance in order to evaluate how changes in rumen microbial community composition may affect the efficiency and quality of beef production (Vasta et al., 2019). Analysis of fatty acids (FAs) and dimethyl acetals (DMAs) originating from rumen microorganisms could be used to understand the effects of a feeding regimen and its influence on rumen microbial metabolism [lipolysis, biohydrogenation (BH) of dietary lipids, synthesis of membrane lipids] (Alves et al., 2013). Previous studies have suggested that the composition of FAs and DMAs in RL may be associated with changes in the rumen microbial community composition resulting from changes in animal diet or feeding regimen (Cappucci et al., 2018; Mannelli et al., 2018) (e.g., grazing vs. feedlot). However, no data are available allowing for comparisons of rumen lipid composition between different rustic cattle breeds maintained on the same farming systems. Moreover, to date, no data are available describing rumen microbial community composition of rustic breeds as affected by different feeding regimens.

The aim of this study was to investigate the patterns of rumen microbial community composition of steers from two cattle breeds (AU and MA steers) as affected by two different feeding regimens (feedlot vs. grazing) and to explore associations with the growth performance of the steers.

## Materials and Methods

### Experimental Design

Forty 4.5-months-old MA and AU (*n* = 20 each) steers with an average body weight of 250 kg were allotted into 2 experimental groups as follows: 10 AU and 10 MA were fed in a feedlot (2,500 m^2^), whereas 10 AU and 10 MA were fed in a grazing (10 ha) system. Each feedlot or pasture area was equally subdivided into 3 lots. Within each rearing system, 10 subjects *per* breed were randomly allotted into 2 subgroups of 3 steers and 1 group of 4 animals, in order to obtain 3 replicates for each treatment and each breed.

The steers in the feedlot were fed grass hay *ad libitum* and concentrated feed (1 kg/100 kg of live weight *per* head and *per* day), whereas grazing animals received grass hay *ad libitum* in addition to the fresh forage available on the pasture that consisted of 62% grass, 17.5% legumes (mainly white clover), and 20.5% other species (Supplementary Table 1). The same kind of concentrate feed used for steers in the feedlot was administered to grazing steers when grass availability was limited [the amount of concentrate was decided monthly on the basis of the individual average daily weight gain (ADG) and was not greater than 1 kg/100 kg of live weight *per* head]. All the animals had free access to water.

Crude protein (CP) ether extract and ash were determined in feeds according to the AOAC (2000) methods. Fiber fractions were analyzed according to Van Soest et al. (1991). Net energy (NE) content of feeds was estimated according to Cornell Net Carbohydrates and Protein System for cattle (Fox et al., 1992). Productivity was evaluated by weighing animals monthly to assess ADG. All steers were slaughtered at 600 kg (between 20 and 22 months old).

All experiments in this study were performed in accordance with the approved guidelines from the European directive 2010/63/UE and DL 4/03/2014 no. 26.

### Sampling of RL and Determination of FAs and DMAs

RL samples were collected from freshly slaughtered steers. The whole rumen content was collected, mixed, and filtered on a sterile cheese cloth (Ramos-Morales et al., 2014) to obtain 200 ml of RL for chemical and microbial analyses. This procedure (i.e., mixing the whole rumen content) ensured the collection of a representative sample of each rumen. The liquid-associated bacteria, and the solid-adherent bacteria associated to the food particles that were not removed by filtering on cheese cloth, were collected. The reproducibility of the results was ensured by sampling 20 animals for each breed. Approximately 2 ml of RL was immediately stored at –80°C for microbial DNA extraction. The rest of the samples were stored at –20°C and then lyophilized (ScanVAC CoolSafe 55-4 lyophilizer, LaboGene ApS DK-3450, Allerød, Denmark) for the analysis of lipid composition.

### Lipid Extraction and Identification of Fatty Acid Methyl Esters and DMAs

Lipids were derivatized by a direct acid/basic double transesterification of freeze-dried RL (Alves et al., 2013). Identification of fatty acid methyl esters (FAMEs) and DMAs was performed by thin-layer chromatography purification of the esterified fraction (Alves et al., 2013). The identification of DMAs was obtained by gas chromatography–mass spectrometry (GC–MS), according to Alves et al. (2013).

The composition of FAs was characterized using a GC2010 Shimadzu gas chromatograph (Shimadzu, Columbia, MD, United States) as previously reported (Alves et al., 2013; Cappucci et al., 2018). Every single FAME was identified through comparison to a standard FAME mixture containing 52 standards (Nu-Chek-Prep Inc., Elysian, MN, United States). Nonanoic acid and nonadecanoic acid were used as internal standards. The identification of the 18:1 isomers was based on a mixture of commercial standards (Supelco, Bellefonte PA, United States) and on the basis of the isomeric profiles (Kramer et al., 2004). Individual FA and DMA profiles were expressed in g/100 g of total FAs and DMAs, respectively.

### DNA Extraction, Sequencing, and Bioinformatics

DNA was extracted from 185 μl of RL by using the Fast DNA Spin for soil kit (MP Biomedicals, Solon, OH, United States) following the manufacturer’s protocol modified as previously reported (Mannelli et al., 2018).

Bacterial 16S rRNA gene amplicons were generated with a double step PCR protocol. The 341F and 806R primers (Bergmann et al., 2011; Zeng et al., 2013) were used for the first round. The same primers with the addition of adaptors and Ion Xpress barcodes were used in the second round. All reactions contained 10 μl of 2× GoTaq Green Master Mix (Promega Corporation, Madison, WI, United States), 2 μl forward primer (1 μM final concentration), 2 μl reverse primer (1 μM final concentration), 1 μl of template DNA, and water for a total volume of 20 μl. The thermocycler program for first PCR consisted of: 95°C for 4 min followed by 25 cycles of 95°C for 30 s, 62°C for 45 s, and 72°C for 2 min, with a final extension at 72°C for 5 min. The second PCR used the same conditions except that an annealing temperature of 65°C and 20 cycles was used. After each round of PCR, amplicons were cleaned in a 96-well plate using Agencourt AMPure XP beads (Beckman Coulter, Inc.). Amplicons were sequenced using an Ion S5^TM^ XL sequencer (Thermo Fisher Scientific) on an Ion 530 chip using 400 bp chemistry.

The UPARSE pipeline (USEARCH 8.1) was used to process the obtained sequences (Edgar, 2010, 2013). The forward primer was removed, the sequences shorter than 350 bp were eliminated, and the remaining sequences were truncated at 350 bp. Low-quality sequences (i.e., sequences with a total expected error > 2) were eliminated. The sequences present only once in the entire dataset (singletons) were removed, and the sequences were grouped into operational taxonomic units (OTUs) at 97% similarity. A representative sequence was selected for each OTU. Representative sequences were classified against SILVA database v138 (Pruesse et al., 2007) using the function assignTaxonomy (confidence 80%) in the DADA2 package, version 1.14.0 (Callahan et al., 2016) in R 3.6.1 (R Core Team, 2020). OTUs with a relative abundance lower than 0.005% in all the samples were removed from the whole dataset. A total of 1,249,694 high-quality sequences were obtained with an average of 31,242 ± 14,662 sequences *per* sample (average ± standard deviation). A randomly rarefied dataset (5,456 sequences per sample—i.e., sequences in the sample with the lowest number of sequences) was generated. The Chao1 index, the ACE index, the Simpson index, and the Shannon diversity index were calculated using the vegan package, version 2.5-6 (Oksanen et al., 2019) in R 3.6.1 (R Core Team, 2020).

### Statistical Analysis

Statistical analysis of RL, FAs, and DMAs was performed by the following general linear model (SAS Institute, Charlotte, NC, United States) (SAS Institute, 2008):

Y_*ijz*_ = μ + B_*i*_ + R_*j*_ + B_*i*_ × R_*j*_ + ε_*ijz*_

Where:

y = observation

μ = overall mean

B_*i*_ = fixed effect of the i-th breed: AU and MA (i: from 1 to 2)

R_*j*_ = fixed effect of the j-th rearing system: pasture and feedlot (j: from 1 to 2)

B_*i*_ × R_*j*_ = interaction effect of the i-th breed and j-th rearing system

ε_*ijz*_ = random error.

In cases of a significant effect for the B_*i*_ × R_*j*_ interaction, a *post-hoc* HSD analysis of Tukey was performed. Probability of significant effect due to experimental factors was fixed at *p* < 0.05.

Data from the characterization of the microbial communities were processed using the vegan package, version 2.5-6 (Oksanen et al., 2019) in R 3.6.1 (R Core Team, 2020). A non-metric multidimensional scaling (NMDS) and a permutational multivariate analysis of variance (PERMANOVA) based on Hellinger transformed OTU abundance data were performed using the metaMDS and the adonis2 functions, respectively. Both the NMDS and the PERMANOVA were performed on the Bray–Curtis dissimilarity index. The taxa with different relative abundances between the conditions (i.e., breed, rearing system, and interaction of breed × rearing system) were identified by a Kruskal–Wallis test and by a *post-hoc* Dunn test with the Benjamini–Hochberg correction for multiple comparison. The Kruskal–Wallis test and the Dunn test were performed to detect significant differences between the conditions for the calculated diversity indexes. Differences were considered significant for *p* < 0.05. The Spearman correlations were performed to identify the bacterial genera correlated to the ADG. Correlations were considered significant for *p* < 0.1.

### Nucleotide Sequence Accession Number

The sequences are available at the National Centre for Biotechnology Information (NCBI), BioProject number PRJNA682716, under the following BioSample accession numbers: SAMN17005974–SAMN17006013.

## Results

### FA and DMA Profiles

The total percentage of saturated fatty acids (SFAs) was higher in the RL from steers reared in the feedlot system (Table 1), with stearic acid (SA, C18:0) being the most relatively abundant, followed by palmitic acid (PA, C16:0) (Table 2). The RL from MA steers had a higher content of PA and a lower content of SA than the RL from steers of the same breed reared in the feedlot, and to the AU steers, regardless of the rearing system (Table 2). The overall content of odd and branched chain FAs (OBCFAs) was higher in the RL from grazing steers of both breeds (Table 1); however, some differences were significant between breeds for specific OBCFA. The RL from AU steers contained a higher percentage of C15:0 *ante* and C16:0 *ante*. In contrast, the RL from MA steers had a higher percentage of C16:0 *iso* and C17:0 *ante* (Table 2). A significant breed × rearing system interaction was found for C17:0 *iso* that was found at a higher percentage in the RL of grazing MA steers. The RL from AU steers contained a higher percentage of odd chain FAs (OCFAs) C13:0, C15:0, C17:0, and C23:0 than the RL from MA steers.

**TABLE 1:** Relative percentage (g/100 g of total FAs + DMAs) of the main classes of fatty acids and dimethyl acetals in the rumen liquor from Aubrac or Maremmana steers maintained in feedlot or on pasture.

	AU		MA		SE	*p*-value		
	Grazing	Feedlot	Grazing	Feedlot		B	R	B × R
SFAs	72.667	76.167	67.674	75.215	1.363	0.045	0.001	0.167
UFAs	21.390	18.683	20.534	17.761	1.309	0.522	0.054	0.981
PUFAs	4.934^b^	4.559^b^	8.254^a^	5.271^b^	0.529	0.001	0.005	0.025
MUFAs	16.456	14.124	12.280	12.490	0.974	0.007	0.307	0.223
PUFAs *n*-6	3.539	3.558	5.452	4.514	0.426	0.003	0.312	0.293
PUFAs *n*-3	1.351^b^	0.961^b^	2.721^a^	0.727^b^	0.220	0.018	<0.001	0.001
*n*-6/*n*-3	2.981^c^	5.168^b^	2.436^c^	8.467^a^	0.765	0.091	<0.001	0.024
*trans* 18:1 tot	10.126	8.197	5.244	5.548	0.755	<0.001	0.312	0.167
VA/*trans* 18:1 tot	0.462	0.401	0.603	0.414	0.034	0.034	0.001	0.081
OCFAs tot	4.719	4.294	4.243	3.236	0.213	0.002	0.003	0.203
OBCFAs tot	6.983	6.131	7.379	5.363	0.370	0.635	<0.001	0.144
OCDMAs tot	2.509	2.071	1.530	0.929	0.142	<0.001	0.001	0.590
DMAs tot	5.221	4.491	5.785	4.213	0.287	0.637	<0.001	0.172
BCFAs tot	4.486^b^	3.907^b^	6.621^a^	4.289^b^	0.394	0.004	0.001	0.041
BCFAs *iso*	1.723	1.533	2.615	1.813	0.144	<0.001	0.003	0.061
BCFAs *ante*	2.766	2.380	3.961	2.454	0.152	0.004	0.003	0.054

**AU, Aubrac; MA, Maremmana; SE, Standard Error; B, breed; R, rearing system; SFAs, saturated fatty acids; UFAs, unsaturated fatty acids; PUFAs, polyunsaturated fatty acids; MUFAs, monounsaturated fatty acids; VA, vaccenic acid; OCFAs, odd chain fatty acids; OBCFAs, odd and branched chain fatty acids; OCDMAs, odd chain dimethyl acetals; DMAs, dimethyl acetals; BCFAs, branched chain fatty acids. a, b, c are the probability of significant effect due to experimental treatment; means within a row with different letters differ (p < 0.05).**

**TABLE 2:** Relative percentage of selected fatty acids (g/100 g of total FAs) in the rumen liquor from Aubrac or Maremmana steers maintained in feedlot or on pasture.

	AU		MA		SE	*p*-value		
	Grazing	Feedlot	Grazing	Feedlot		B	R	B × R
C12:0	0.340	0.254	0.197	0.195	0.046	0.045	0.370	0.391
C13:0 *iso*	0.084	0.073	0.067	0.061	0.009	0.144	0.380	0.800
C13:0 *ante*	0.013	0.015	0.017	0.012	0.002	0.853	0.308	0.085
C13:0	0.113	0.124	0.045	0.045	0.013	<0.001	0.691	0.696
C14:0 *iso*	0.326	0.247	0.316	0.245	0.029	0.825	0.018	0.894
C14:0	1.495	1.701	1.050	0.964	0.113	<0.001	0.616	0.224
C15:0 *iso*	0.641	0.609	0.771	0.549	0.048	0.492	0.016	0.068
C15:0 *ante*	1.964	1.786	1.419	1.156	0.097	<0.001	0.037	0.678
C15:0	1.466	1.323	1.336	1.003	0.089	0.021	0.015	0.313
C16:0 *iso*	0.361	0.273	0.956	0.647	0.057	<0.001	0.002	0.074
C16:0 *ante*	0.463	0.284	0.297	0.104	0.066	0.017	0.011	0.921
C16:0	17.719^*b*^	16.485^*b*^	23.766^a^	18.795^*b*^	0.722	<0.001	<0.001	0.019
C17:0 *iso*	0.335^*b*^	0.294^*b*^	0.624^a^	0.352^*b*^	0.047	0.001	0.003	0.026
C16:1 *cis*7	0.116	0.107	0.056	0.059	0.009	<0.001	0.714	0.498
C16:1 *cis*9	0.150	0.138	0.206	0.090	0.060	0.946	0.310	0.415
C17:0 *ante*	0.478	0.405	1.089	0.653	0.093	<0.001	0.013	0.072
C17:0	0.764	0.717	0.678	0.575	0.038	0.007	0.070	0.489
C18:0 *iso*	0.072	0.109	0.047	0.051	0.023	0.092	0.403	0.487
C18:0	48.192^*b*^	53.365^a^	37.282^c^	51.652^a^	1.994	0.005	<0.001	0.035
C18:1 *trans*6–8	0.694	0.841	0.264	0.371	0.135	0.003	0.375	0.890
C18:1 *trans*9	0.378	0.451	0.197	0.247	0.066	0.009	0.375	0.870
C18:1 *trans*10	2.407	2.027	0.597	0.993	0.400	0.002	0.985	0.363
C18:1 *trans*11	4.889	3.385	3.375	2.424	0.459	0.015	0.015	0.570
C18:1 *trans*12	0.786^a^	0.686^a^	0.437^*b*^	0.622^a^	0.044	<0.001	0.367	0.004
C18:1 *cis*9	4.836	4.312	6.290	5.968	0.310	<0.001	0.202	0.758
C18:1 *trans*15	0.684^a^	0.538^*b*^	0.291^c^	0.491^*b*^	0.040	<0.001	0.531	< 0.001
C18:1 *cis*11	0.984	1.098	0.540	0.658	0.095	<0.001	0.253	0.983
C18:1 *cis*12	0.353^a^	0.337^a^	0.216^*b*^	0.323^a^	0.021	0.001	0.044	0.007
C18:1 *cis*13	0.038	0.028	0.031	0.028	0.003	0.260	0.076	0.265
C18:1 *trans*16	0.834^a^	0.642^*b*^	0.400^c^	0.643^*b*^	0.045	<0.001	0.598	< 0.001
C19:1 *trans*7	0.096	0.075	0.062	0.059	0.009	0.012	0.188	0.338
C18:2 *n*-6	3.632	3.635	5.673	4.632	0.454	0.003	0.284	0.281
C20:0	0.806^a^	0.824^a^	0.896^a^	0.717^*b*^	0.036	0.824	0.040	0.013
C20:1 *cis*11	0.102	0.109	0.073	0.063	0.014	0.013	0.902	0.547
C18:3 *n*-3	0.959	0.517	1.340	0.369	0.170	0.518	<0.001	0.148
C18:2 *cis*9*trans*11	0.046	0.042	0.085	0.032	0.013	0.283	0.047	0.081
C21:0	0.046	0.044	0.057	0.044	0.008	0.514	0.357	0.456
C18:4 *n*-3	0.151^*b*^	0.173^*b*^	0.255^a^	0.078^c^	0.043	0.929	0.094	0.034
C22:0	0.513^*b*^	0.465^*b*^	0.678^a^	0.416^*b*^	0.037	0.142	<0.001	0.009
C20:3 *n*-6	0.020	0.021	0.045	0.030	0.005	0.001	0.161	0.095
C23:0	0.221	0.155	0.111	0.101	0.026	0.005	0.179	0.312
C24:0	0.606	0.509	0.724	0.525	0.040	0.123	0.001	0.236
C22:4 *n*-6	0.082	0.066	0.086	0.057	0.007	0.702	0.005	0.403
C22:5 *n*-3	0.315^*b*^	0.317^*b*^	1.300^a^	0.316^*b*^	0.111	<0.001	<0.001	<0.001
Unknown	0.405^c^	0.377^c^	5.419^a^	2.473^*b*^	0.332	<0.001	<0.001	<0.001

**AU, Aubrac; MA, Maremmana; SE, Standard Error; B, breed; R, rearing system; Unknown, sum of three unknown peaks. a, b, c are the probability of significant effect due to experimental treatment; means within a row with different letters differ (p < 0.05).**

The overall percentage of polyunsaturated fatty acids (PUFAs) was significantly higher in RL from grazing MA steers, as a consequence of the significant breed × rearing system interaction (Table 1). Considering the PUFA *n*–3 FA, the interaction was significant for C22:5 *n*–3 (*p* < 0.001) and for C18:4 *n*–3 (*p* = 0.034). Linoleic acid (C18:2 *n*–6) was the most relatively abundant PUFA, and it was found at higher percentage in RL from MA steers, irrespective of the rearing system, together with *n*–6 (C20:3 *n*–6, C22:4 *n*–6) PUFAs. The *cis* and *trans* C18:1 isomers were differentially distributed in RL according to the breed and feeding strategy. Vaccenic acid (VA, C18:1 *trans*11) was the C18:1 *trans* isomer with the highest concentration in the RL, regardless of the breed and the rearing system. The highest concentration of VA was detected in the RL from AU steers, whereas the C18:1 *cis*9, the most abundant among the C18:1 *cis* isomers and monounsaturated fatty acids (MUFAs), was contained at the greatest concentration in the RL from MA steers. The content of C18:1 *trans*6–8, C18:1 *trans*9, C18:1 *trans*10, C18:1 *trans*12, C18:1 *trans15*, C18:1 *trans*16, C18:1 *cis*11, and C18:1 *cis*12 was higher in the RL from AU steers. The interaction of breed × rearing system was significant in a few cases: C18:1 *trans*12, C18:1 *trans*15, C18:1 *cis*12, and C18:1 *trans*16, being the FAs found at the lowest percentage in the RL from MA grazing steers. Grazing activity resulted in a significant increase of α-linolenic acid (α-LNA, C18:3 *n*–3) in the RL, especially for the MA steers that had the highest amount of α-LNA in their RL (*p* = 0.148). A decrease of the *n*–6/*n*–3 ratio in the RL from grazing steers was also observed, regardless of the breed. The concentration of conjugated linoleic acid (CLA) did not vary across treatments (Table 2).

The total content of DMA in RL did not vary between breeds but was higher in the RL from grazing steers (Table 1). Nineteen different DMAs were identified in the RL (Table 3). The most abundant DMA was DMA C16:0, followed by DMA C15:0 *iso* for AU steers and DMA C14:0 for MA steers. Overall, 16 DMAs changed significantly in their content according to the breed factor, and only DMA C15:0 *ante*, DMA C17:0, and DMA C18:1 *trans*11 did not significantly differ between the two breeds. DMA C15:0 *iso*, DMA C16:1, and DMA C18:1 *cis*9 were significantly more abundant in the RL from AU steers, whereas the relative percentages of the other DMAs were higher in the RL from MA steers. In four cases (DMA C14:0, DMA C15:0, DMA C18:1 *cis*11, and DMA C18:1 *cis*12), the interaction breed × rearing system was significant. The rearing system showed a significant effect only for DMA C18:1 *cis*11. Overall, the content of odd chain DMAs and of branched chain DMAs was higher in the RL from AU steers, mainly due to the higher content of DMA C15:0 *iso* (Table 3).

**TABLE 3:** Relative percentage of dimethyl acetals (g/100 g of total DMAs) in the rumen liquor from Aubrac or Maremmana steers maintained in feedlot or on pasture.

	AU		MA		SE	*p*-value		
	Grazing	Feedlot	Grazing	Feedlot		B	R	B × R
DMA C13:0	0.785	0.995	1.104	1.610	0.141	<0.001	0.142	0.366
DMA C13:0 *iso*	0.548	0.540	1.171	0.599	0.171	0.025	0.113	0.113
DMA C14:0	5.454^c^	7.845^*b*^	10.114^a^	9.735^a^	0.536	<0.001	0.097	0.012
DMA C14:0 *iso*	6.837	6.331	7.865	8.017	0.919	0.017	0.794	0.754
DMA C15:0	5.180^c^	5.720^c^	7.021^a^	6.046^*b*^	0.238	<0.001	0.321	0.003
DMA C15:0 *ante*	2.998	2.788	3.697	3.180	0.644	0.503	0.568	0.781
DMA C15:0 *iso*	24.628	23.026	6.261	5.389	1.008	<0.001	0.245	0.782
DMA C16:0	30.499	30.682	40.405	38.091	1.274	<0.001	0.370	0.267
DMA C16:1	1.920	1.765	0.796	0.964	0.185	<0.001	0.967	0.423
DMA C16:0 *iso*	1.649	1.538	4.127	4.444	0.299	<0.001	0.814	0.547
DMA C17:0	0.426	0.458	0.320	0.466	0.075	0.455	0.286	0.517
DMA C17:1	1.439	1.140	1.867	1.460	0.258	0.044	0.179	0.805
DMA C17:0 *ante*	0.615	0.755	1.393	1.449	0.171	<0.001	0.625	0.772
DMA C17:0 *iso*	0.613	0.699	1.014	0.793	0.102	0.003	0.537	0.163
DMA C18:0	2.853	3.214	3.348	3.382	0.159	<0.001	0.290	0.269
DMA C18:1 *cis*11	2.584^*b*^	2.338^*b*^	1.954^*b*^	4.299^a^	0.368	0.012	0.012	0.002
DMA C18:1 *cis*12	0.870^c^	0.464^*d*^	1.055^*b*^	1.752^a^	0.222	<0.001	0.568	0.024
DMA C18:1 *cis*9	8.452	7.921	4.793	6.333	0.673	<0.001	0.508	0.162
DMA C18:1 *trans*11	1.650	1.779	1.694	1.991	0.192	0.109	0.321	0.717

**DMA, dimethyl acetal; AU, Aubrac; MA, Maremmana; SE, Standard Error; B, breed; R, rearing system. a, b, c are the probability of significant effect due to experimental treatment; means within a row with different letters differ (p < 0.05).**

### Taxonomic Composition of the Bacterial Communities

**FIGURE 1:**
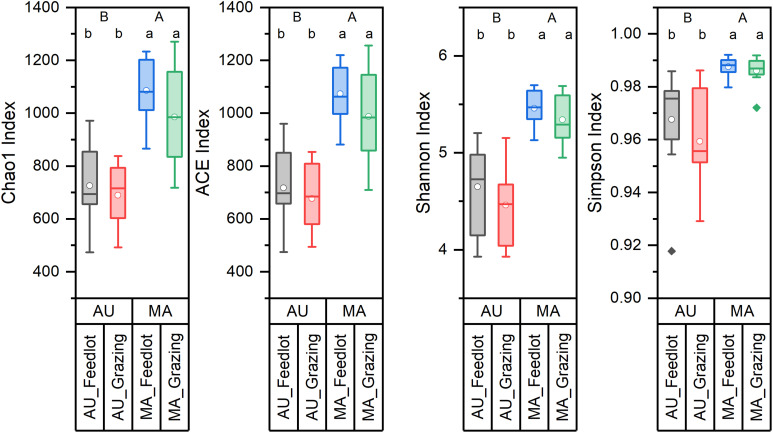
Diversity indices of the rumen microbiota of Aubrac (AU) and Maremmana (MA) breeds in the two rearing systems (feedlot and grazing). Uppercase letters (A and B) show significant differences (*p* < 0.05) between breeds. Lowercase letters (a and b) show significant differences (*p* < 0.05) due to the interaction breed × rearing system. The biodiversity is lower in the bacterial communities in the rumen of AU steers.

Bacterial communities in the rumen of AU and MA steers reared in two different systems were characterized by high-throughput sequencing of 16S rRNA gene amplicons. Rarefaction curves (Supplementary Figure 1), obtained by plotting the number of OTUs vs. the number of sampled sequences, indicated that the depth of the sampling was enough to describe the biodiversity within the dataset. Chao1, ACE, Shannon, and Simpson diversity indexes (Figure 1) were calculated and clearly showed a difference (in terms of both richness and evenness) between the rumen bacterial communities in the two breeds. Conversely, no differences were observed in the diversity for the two rearing systems.

**FIGURE 2:**
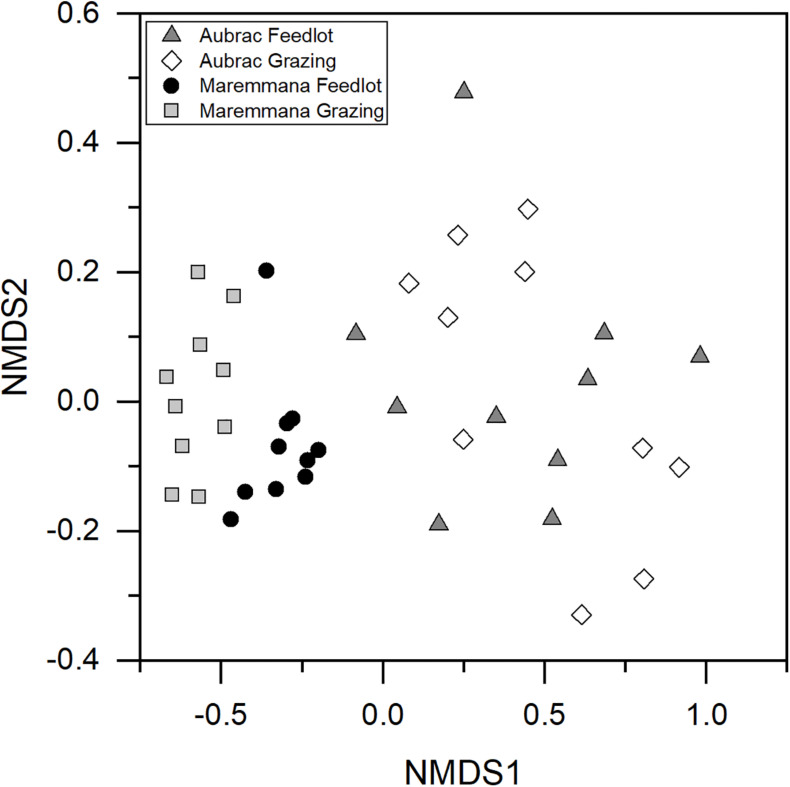
Non-metric multidimensional scaling plot based on Hellinger transformed OTU abundance data. The bacterial communities selected in the rumen of the two breeds reared in different conditions are different.

An NMDS plot showed that the composition of the microbial communities was affected by the breed (Figure 2), an observation confirmed by PERMANOVA (*R*^2^ = 0.28, *p* < 0.001). Furthermore, despite the diversity within the microbial communities in the two rearing systems not being different, a significant difference in the composition (i.e., in the structure) of the bacterial communities was observed for the rearing system (*R*^2^ = 0.04, *p* = 0.021) and for the interaction breed × rearing system (*R*^2^ = 0.04, *p* = 0.029).

**FIGURE 3:**
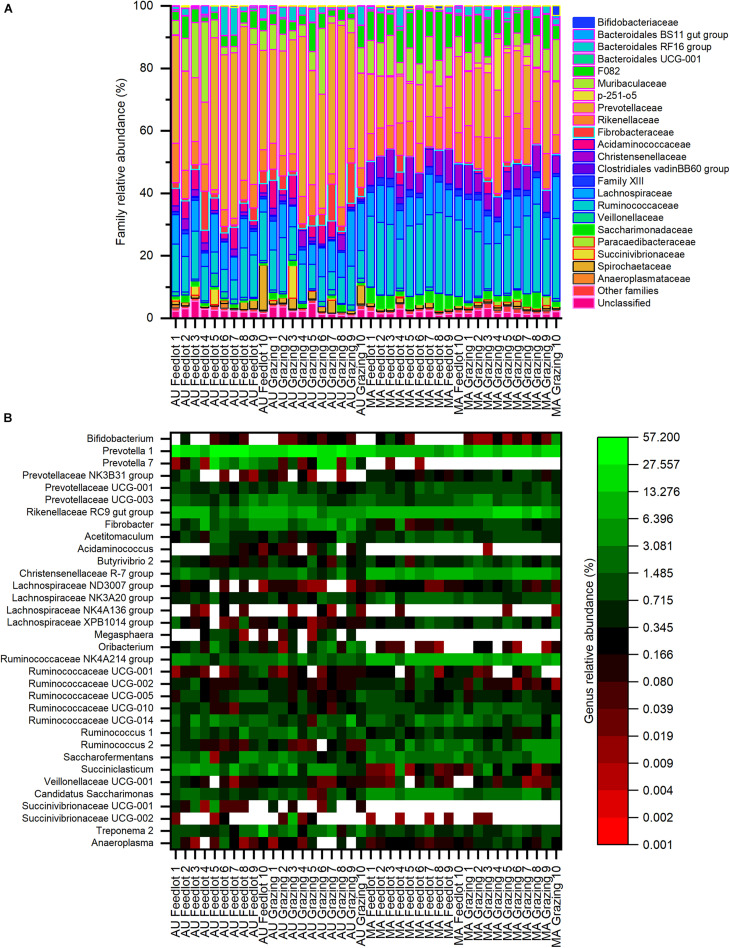
Composition of bacterial communities at the **(A)** family and **(B)** genus levels for the Aubrac (AU) and Maremmana (MA) breeds in the two rearing systems (feedlot and grazing). Only the genera with a relative abundance of at least 1% in at least one sample are reported. Within the whole dataset, the most relatively abundant families were Prevotellaceae, Ruminococcaceae, and Lachnospiraceae, whereas the most relatively abundant genus was *Prevotella*.

Considering the whole dataset, the family Prevotellaceae (phylum *Bacteroidetes*) was the most relatively abundant (average relative abundance ∼38%, with a range between ∼10 and ∼58%), followed by the families Ruminococcaceae and Lachnospiraceae (phylum *Firmicutes*), with an average relative abundance of ∼11% (range between ∼6 and ∼28%) and ∼8% (range between ∼4 and ∼18%), respectively (Figure 3A). At the genus level (Figure 3B), the most relatively abundant groups were *Prevotella* 1 (phylum *Bacteroidetes*, average ∼27%, with a range between ∼6 and ∼57%) and Rikenellaceae RC9 gut group (phylum *Bacteroidetes*, average ∼7%, with a range between ∼1 and ∼18%), followed by the genus *Succiniclasticum* (phylum *Firmicutes*, average ∼4%, with a range between < 1 and ∼9%).

Only 16 families showed an average relative abundance of 1%, or higher, in at least one group (i.e., AU grazing, AU feedlot, MA grazing, MA feedlot— Supplementary Table 2). Twelve families showed significantly different relative abundances between the two breeds. In particular, the families Prevotellaceae, Fibrobacteraceae, Acidaminococcaceae, Veillonellaceae, and Succinivibrionaceae were more relatively abundant in the AU breed, whereas the families F082, p-251-o5, Christensenellaceae, Family XIII, and Ruminococcaceae were more abundant in the MA breed. Furthermore, the relative abundance of the family Rikenellaceae was higher (∼12%) in the MA steers reared in grazing conditions than in the AU steers reared in both pasture and feedlot, whereas the relative abundance of this family in MA steers reared in feedlot was intermediate (Supplementary Table 2). Similarly, the family Saccharimonadaceae showed a higher relative abundance in the rumen of MA steers reared in the feedlot than of AU steers, regardless of the rearing conditions, and the relative abundance in the RL from MA steers reared in pasture was intermediate (Supplementary Table 2).

Twenty genera with an average relative abundance of 1%, or higher, in at least one group were observed (Supplementary Table 3). The genera *Prevotella* (*Prevotella* 1 and *Prevotella* 7), *Fibrobacter*, *Oribacterium*, and *Succiniclasticum* were more relatively abundant in the rumen of AU steers, whereas the genera *Acetitomaculum*, *Saccharofermentans*, and some members of the families Christensenellaceae and Ruminococcaceae (Christensenellaceae R-7 group, Ruminococcaceae NK4A214 group, Ruminococcaceae UCG-010, *Ruminococcus* 2) were more relatively abundant in the rumen of MA steers, regardless of the rearing system. Four genera had a different relative abundance not only considering the breed but also considering the interaction breed × rearing system: the Lachnospiraceae NK3A20 group and *Candidatus Saccharimonas* showed a significantly higher relative abundance in the rumen microbial communities in MA steers reared in the feedlot than in AU steers reared in both grazing and feedlot systems (Supplementary Table 3). Similarly, the Rikenellaceae RC9 gut group had a higher relative abundance in MA steers in pasture conditions than in AU steers in both rearing systems, whereas the genus *Ruminococcus* 1 showed the opposite and had a higher relative abundance in the rumen of AU grazing steers than of MA grazing steers (Supplementary Table 3).

### Correlation Between Bacterial Taxa and Growth Performance

**FIGURE 4:**
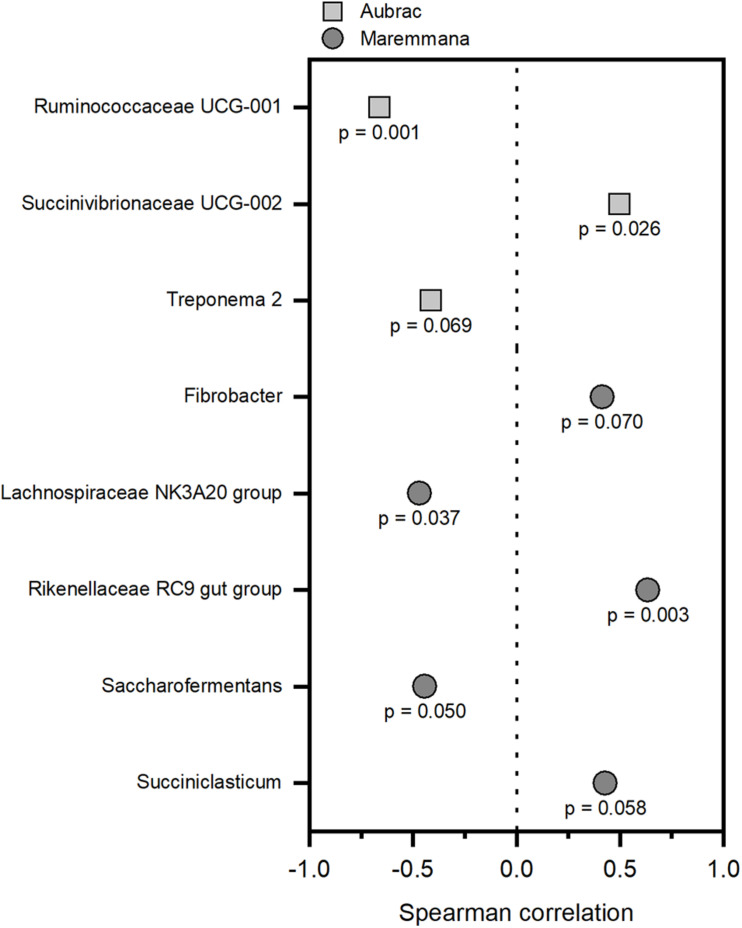
Correlations between the rumen bacteria and the average daily weight gain of the Aubrac cattle and the Maremmana cattle. Only significant correlations (*p* < 0.1) were reported. Correlations were calculated independently for each breed, and only the genera with a relative abundance of 1%, or higher, in at least one sample were considered.

The ADG values were less than 1 kg *per* head and *per* day and were higher for the AU steers than for the MA steers (Supplementary Table 4). To correlate the bacterial groups to growth performance, Spearman correlations were obtained independently for each breed. Only genera with a relative abundance of at least 1% in at least one sample were considered for each breed. At the genus level, different bacterial genera showed a significant correlation with growth performance (*p* < 0.1) in the two breeds (Figure 4). The groups Ruminococcaceae UCG-01, *Treponema* 2 (AU), Lachnospiraceae NK3A20 group, and *Saccharofermentans* (MA) were negatively correlated to growth performance. Conversely, the relative abundance of Succinivibrionaceae UCG-002 (AU), Rikenellaceae RC9 gut group, *Fibrobacter*, and *Succiniclasticum* (MA) increased in accordance to growth performance.

## Discussion

AU and MA steers were reared under two different conditions (i.e., grazing and feedlot). The ADG was recorded, and at the end of the rearing period, samples of RL were collected to analyze the FA and DMA profiles and to characterize the bacterial communities. The diet is a key factor that modulates the microbial community and, consequently, the BH of FAs in the rumen (Vasta et al., 2019). However, the breed can also influence the rumen microbiota, methane emissions, and feed efficiency (Paz et al., 2016; Roehe et al., 2016; Li et al., 2019a,b). Hence, the presence of functional FAs in animal products can be influenced both by host genetics and by the feeding strategy (Vasta et al., 2019).

In our study, SA and PA were the most relatively abundant FAs in all the samples, which are consistent with the findings from several authors (Cersosimo et al., 2016; Buccioni et al., 2017; Cappucci et al., 2018; Mannelli et al., 2018). FA profiles, however, showed several differences in the RL collected from the two breeds. The SA content was lower in the RL from grazing animals than in the RL from animals fed in feedlot, with the lowest percentage recorded in grazing MA steers. The feeding strategy based on pasture increased the percentage of C18:3 *n*–3. This observation can be explained since this FA is a structural FA whose presence can be observed in fresh herbs. This finding (i.e., higher percentage of C18:3 *n*–3 in RL of grazing steers, linked to lower concentration of SA in RL of MA steers reared in pasture) suggests less intensive BH in the RL of grazing MA steers. A different modulation of the BH pathway in the two breeds, regardless of the feeding strategy, is confirmed also by the amount of C18:1 *trans* isomers that is higher in the RL of AU steers than of MA steers. These FAs are important markers of rumen microbial metabolism and are produced not only by hydrogenation of PUFAs but also by isomerization of MUFAs (Mosley et al., 2002). A feeding strategy based on grazing is able to modify the FAs profile in the rumen (Collomb et al., 2002). Grazing animals can select plant species, or specific components of forage plants, with different secondary metabolites or C18:3 *n*3 contents. This can lead to a different intake of such molecules. MA and AU could have a different feeding behavior and could be characterized by a different rate of passage of forage in the rumen due to their physiology. These factors could have induced changes in the profile of the microbial community, thus affecting the rate of BH (Lee et al., 2003; Cabiddu et al., 2010; Vasta et al., 2019). The effect of polyphenols on the bacterial communities involved in BH in the rumen of animals fed fresh grass has been described in the literature and can explain the reduced disappearance of PUFAs (Vasta et al., 2019). The accumulation of PUFAs can further influence the microbial activity due to their antibacterial properties, leading to additional changes in the BH pathway (Jenkins, 1994; Chilliard et al., 2001). In this trial, the animals grazed on a pasture composed of grass and legumes and a previous study showed that the proportion of C18 PUFAs by-passing the rumen and entering the duodenum was higher in steers fed legume silage than in steers fed grass silage (Lee et al., 2003). Furthermore, a negative relationship between the content of polyphenols in several legumes and the trend of BH of C18:3 *n*3 during *in vitro* fermentation has been demonstrated (Cabiddu et al., 2010). Moreover, differences in the amount of C18:1 *cis*9 and C18:3 *cis*9 *cis*12 *cis*15 (higher in MA steers) and VA (higher in AU steers) could be related to a selective action in protecting *cis*9 double bound from hydrogenation in MA steers and in lowering *trans*11 hydrogenation in AU steers, which can be influenced by the different compositions of microbial communities between the two breeds.

OBCFAs are largely present in bacterial membranes (Kaneda, 1991) and are therefore considered microbial markers in the rumen ecosystem (Vlaeminck et al., 2006; Bessa et al., 2009). The results of the present study provide evidence that the total concentration of OBCFA was higher in the RL of grazing steers and, in particular, in MA grazing steers. Other authors observed that the proportion of even-chain saturated FA decreased, and that of BCFA increased with increasing forage content (Bas et al., 2003). In our study, grazing regimen increased the concentration of *iso* branched chain FAs, in particular 14:0 *iso*,15:0 *iso*, 16:0 *iso*, and 17:0 *iso*. An increase of amylolytic bacteria can increase *ante iso* and linear OCFAs, whereas an increase in cellulolytic bacteria can result in a higher amount of *iso* FAs (Vlaeminck et al., 2006). Our data suggest that that the feeding regimen of MA grazing steers was probably richer in forage than that of AU grazing steers, possibly due to more intense grazing activity by MA steers. Although the individual consumption of forage and concentrate was not recorded during the experiment, the total amount of concentrate consumed by the grazing MA steers as a group was lower than that recorded by the grazing AU steers (data not shown), providing indirect confirmation of this hypothesis.

Microbial plasmalogens (detected as DMAs) have been used to compare microbial communities in RL when different diets were provided (Mannelli et al., 2019). Several authors observed that specific DMAs are associated with specific bacterial taxa under certain conditions, indicating that plasmalogen lipid profiles can be considered as a tool for the identification of the effect that a change in environmental conditions can induce on a microbial community (Miyagawa, 1982; Minato et al., 1988). In this study, DMA C16:0 was the most abundant in all groups, which was in agreement with previous results (Alves et al., 2013). The main factor affecting DMA profile in this study was the breed, and the results were confirmed by the 16S rRNA gene amplicon sequencing.

Differences in the composition of the bacterial communities in RL between the two breeds were observed by high-throughput sequencing of 16S rRNA gene amplicons. Bacterial diversity was higher in the rumen communities of MA steers than of AU steers. AU steers, however, showed higher ADG. This observation is in agreement with previous findings in which the more efficient animals were characterized by a lower biodiversity of the microbial taxa and of the metabolic pathways active in the rumen (Shabat et al., 2016). The most relatively abundant groups were the same in the two breeds (i.e., Prevotellaceae, Ruminococcaceae, and Lachnospiraceae at the family level and *Prevotella* 1, Rikenellaceae RC9 gut group, and *Succiniclasticum* at the genus level), regardless of the rearing system. The genus *Prevotella* is a dominant member of the bacterial community in the rumen (Stevenson and Weimer, 2007; Henderson et al., 2015; Mannelli et al., 2019) and is among the bacterial groups of the so-called rumen core microbiota (Henderson et al., 2015), along with members of the families Ruminococcaceae and Lachnospiraceae (e.g., microorganisms of the genera *Ruminococcus* and *Butyrivibrio*) (Henderson et al., 2015; Mannelli et al., 2019). The importance of these bacteria in the ecology of rumen communities and their key role in the degradation of cellulose, pectin, and hemicellulose has been also well documented (Kabel et al., 2011; Seshadri et al., 2018).

The presence of bacteria belonging to the genus *Succiniclasticum* is widely described in rumen communities (Holman and Gzyl, 2019; Auffret et al., 2020). Members of this genus are involved in the conversion of succinate to propionate (van Gylswyk, 1995). Furthermore, the abundance of propionate-producing bacteria (i.e., *Succiniclasticum* spp.) is positively correlated to animal feed efficiency (Auffret et al., 2020; Clemmons et al., 2020), since propionate is the main precursor of glucogenesis (Shabat et al., 2016). This is also confirmed by our study, in which a positive correlation was observed between the genus *Succiniclasticum* and the growth performance in the MA steers, indicating that the relative abundance of this genus in the rumen increased according to the ADG. Other than the genus *Succiniclasticum*, the genus *Fibrobacter* was also correlated with the growth of MA steers in our study. Members of the genus *Fibrobacter* are indeed involved in the conversion of cellulose to succinate that can be used as a substrate for the production of propionate by the genus *Succiniclasticum* (Holman and Gzyl, 2019). Furthermore, these two genera (i.e., *Fibrobacter* and *Succiniclasticum*) were more abundant in the rumen of AU steers (which showed higher ADG) than of MA steers, despite the fact that their presence in AU steers was not correlated to the growth performance, suggesting the occurrence of different fermentation patterns in the two breeds. The presence of the Rikenellaceae RC9 gut group (family Rikenellaceae) has been widely observed in the gastrointestinal tract of several ruminants (Holman and Gzyl, 2019; Tong et al., 2020). The Rikenellaceae RC9 gut group was positively correlated with growth performance in MA steers and was found to be more relatively abundant in the MA steers (in particular when this breed is reared under grazing conditions) than in the AU steers. These microorganisms can be involved in the production of short chain FAs (Holman and Gzyl, 2019) and in the scavenging of H_2_, lowering methanogenesis rates (Tong et al., 2020). This reinforces the hypothesis that an increase in growth performance can be correlated to the production of propionate, which is not only a glucogenic precursor but also a sink of H_2_ leading to more efficient energy recovery.

Two bacterial taxa (i.e., Lachnospiraceae NK3A20 group—family Lachnospiraceae and *Saccharofermentans*—family Ruminococcaceae) were negatively correlated with growth performance in MA steers. This result is consistent with the observation of another study in which microorganisms assigned to the family Lachnospiraceae and to the genus *Saccharofermentans* were dominant in the rumen of dairy cows with low milk yields (Sofyan et al., 2019). Despite the fact that *Saccharofermentans* and Lachnospiraceae spp. are involved in the production of short chain FAs, the negative correlation of their relative abundance with growth performance of MA steers could suggest that the pathways that involve these groups are less efficient in this breed (i.e., MA), than the pathways that involve the genus *Succiniclasticum* and the Rikenellaceae RC9 gut group.

Correlation with ADG in the AU steers differed from those in the MA steers, in accordance with our previous observations, which clearly showed that the main driving factor that influenced the microbial communities in this work was the breed. A significant effect of breed on the rumen microbiota was reported in Holstein and Jersey cows (Paz et al., 2016) and in Angus, Charolais, and Kinsella composite hybrid cattle (Li et al., 2019a). Our study extends this observation to MA and AU cattle, which are rustic breeds, that are well-adapted to extensive farming conditions, with potential advantages in terms of sustainability of animal production. The only similarity that was observed in the two breeds is in regard to members of the family Ruminococcaceae, since in the rumen of AU steers, an uncultured member of this family (i.e., Ruminococcaceae UCG-001) had a negative correlation with performance, in analogy to the genus *Saccharofermentans* in the rumen of MA steers. Further, the genus *Treponema* had a negative correlation with growth performance in AU steers. In the literature, the role of the genus *Treponema* in the rumen is still controversial, with both negative and positive correlations to animal performance reported. Negative correlations are usually explained by the potential pathogenic role of this microorganism (Mao et al., 2015), whereas positive correlations are explained by the fact that some species within this genus exhibit pectinolytic activity (Auffret et al., 2020). The only microbial group with a positive correlation with growth performance in the rumen of AU steers comprised an uncultured member of the family Succinivibrionaceae (i.e., Succinivibrionaceae UCG-002). These microorganisms are involved in the production of succinate and acetate, and their presence has been linked to low methane emissions from cows (Holman and Gzyl, 2019). Furthermore, a higher abundance of the genus *Succinivibrio* was observed in the rumen of cattle with higher feed efficiency (Auffret et al., 2020). Our observations suggest that different microbial groups can be correlated with animal performance in the two breeds, but that the microbial pathways that influence the performance could be the same (i.e., short chain FAs production and the presence of H_2_ sinks as an alternative to methanogenesis). This evidence reinforces the hypothesis that different bacterial groups can have similar functions in the rumen ecosystem. Therefore, future studies should be focused on the impact that specific metabolic functions and pathways have on the rumen microbiota and on animal traits.

In this study, the microbial communities and the FA profiles in the rumens of two breeds of rustic cattle reared under different conditions were characterized. While the rearing system shaped the rumen microbiome structure, the main factor that influenced the bacterial communities was the cattle breed, indicating for the first time that host genetics plays an important role also in rustic cattle. This aspect is of particular interest since rustic cattle, such as AU and MA, are well suited to extensive farming in harsh environments, such as the Mediterranean area. Furthermore, while microorganisms involved in the production of propionate seem to have an influence on growth performance, different taxa had a positive influence on the weight gain in the two breeds, reinforcing once again the hypothesis that host genetics can influence the rumen microbiome.

## Data Availability Statement

The datasets presented in this study can be found in online repositories. The names of the repository/repositories and accession number(s) can be found below: https://www.ncbi.nlm.nih.gov/, BioProject number PRJNA682716, BioSample accession numbers SAMN17005974-SAMN17006013.

## Ethics Statement

Ethical review and approval was not required for the animal study because all experiments in this study were performed in accordance with the approved guidelines from the European directive 2010/63/UE and DL 4/03/2014 n 26.

## Author Contributions

MD, FC, AB, AS, CV, and MM conceived and designed the research. MD and FC wrote the manuscript. MD performed the bioinformatic analysis. FC, AC, and AS performed the field sampling. MD, GC, and MM performed the statistical analysis. LC and FC performed the chemical analyses. BM and JV performed the amplicons sequencing. AB, CV, and MM reviewed the manuscript. All authors read and approved the submitted version.

## Conflict of Interest

The authors declare that the research was conducted in the absence of any commercial or financial relationships that could be construed as a potential conflict of interest.

## References

[B1] Alves S. P. Santos-Silva J. Cabrita A. R. J. Fonseca A. J. M. Bessa R. J. B. (2013). Detailed dimethylacetal and fatty acid composition of rumen content from lambs fed lucerne or concentrate supplemented with soybean oil. *PLoS One* 8:e58386. 10.1371/journal.pone.0058386 23484024 PMC3587585

[B2] AOAC (2000). *Official Methods of Analysis*, 17th Edn. Arlington, VA: AOAC.

[B3] Auffret M. D. Stewart R. D. Dewhurst R. J. Duthie C.-A. Watson M. Roehe R. (2020). Identification of microbial genetic capacities and potential mechanisms within the rumen microbiome explaining differences in beef cattle feed efficiency. *Front. Microbiol.* 11:1229. 10.3389/fmicb.2020.01229 32582125 PMC7292206

[B4] Bas P. Archimède H. Rouzeau A. Sauvant D. (2003). Fatty acid composition of mixed-rumen bacteria: effect of concentration and type of forage. *J. Dairy Sci.* 86 2940–2948. 10.3168/jds.S0022-0302(03)73891-0 14507030

[B5] Bergmann G. T. Bates S. T. Eilers K. G. Lauber C. L. Caporaso J. G. Walters W. A. (2011). The under-recognized dominance of Verrucomicrobia in soil bacterial communities. *Soil Biol. Biochem.* 43 1450–1455. 10.1016/j.soilbio.2011.03.012 22267877 PMC3260529

[B6] Bessa R. J. B. Maia M. R. G. Jerónimo E. Belo A. T. Cabrita A. R. J. Dewhurst R. J. (2009). Using microbial fatty acids to improve understanding of the contribution of solid associated bacteria to microbial mass in the rumen. *Anim. Feed Sci. Technol.* 150 197–206. 10.1016/j.anifeedsci.2008.09.005

[B7] Bongiorni S. Gruber C. E. M. Chillemi G. Bueno S. Failla S. Moioli B. (2016). Skeletal muscle transcriptional profiles in two Italian beef breeds, Chianina and Maremmana, reveal breed specific variation. *Mol. Biol. Rep.* 43 253–268. 10.1007/s11033-016-3957-3 26896938

[B8] Buccioni A. Pallara G. Pastorelli R. Bellini L. Cappucci A. Mannelli F. (2017). Effect of dietary chestnut or quebracho tannin supplementation on microbial community and fatty acid profile in the rumen of dairy ewes. *Biomed. Res. Int.* 2017:4969076. 10.1155/2017/4969076 29457028 PMC5804114

[B9] Cabiddu A. Salis L. Tweed J. K. S. Molle G. Decandia M. Lee M. R. F. (2010). The influence of plant polyphenols on lipolysis and biohydrogenation in dried forages at different phenological stages: in vitro study. *J. Sci. Food Agric.* 90 829–835. 10.1002/jsfa.3892 20355119

[B10] Callahan B. J. McMurdie P. J. Rosen M. J. Han A. W. Johnson A. J. A. Holmes S. P. (2016). DADA2: high-resolution sample inference from Illumina amplicon data. *Nat. Methods* 13 581–583. 10.1038/nmeth.3869 27214047 PMC4927377

[B11] Cappucci A. Alves S. P. Bessa R. J. B. Buccioni A. Mannelli F. Pauselli M. (2018). Effect of increasing amounts of olive crude phenolic concentrate in the diet of dairy ewes on rumen liquor and milk fatty acid composition. *J. Dairy Sci.* 101 4992–5005. 10.3168/jds.2017-13757 29525320

[B12] Cersosimo L. M. Bainbridge M. L. Wright A.-D. G. Kraft J. (2016). Breed and lactation stage alter the rumen protozoal fatty acid profiles and community structures in primiparous dairy cattle. *J. Agric. Food Chem.* 64 2021–2029. 10.1021/acs.jafc.5b05310 26752342

[B13] Chilliard Y. Ferlay A. Doreau M. (2001). Effect of different types of forages, animal fat or marine oils in cow’s diet on milk fat secretion and composition, especially conjugated linoleic acid (CLA) and polyunsaturated fatty acids. *Livest. Prod. Sci.* 70 31–48.

[B14] Clemmons B. A. Powers J. B. Campagna S. R. Seay T. B. Embree M. M. Myer P. R. (2020). Rumen fluid metabolomics of beef steers differing in feed efficiency. *Metabolomics* 16:23. 10.1007/s11306-020-1643-x 31989305

[B15] Collomb M. Ueli B. Sieber R. Jeangros B. Bosset J.-O. (2002). Correlation between fatty acids in cows’ milk fat produced in the lowlands, mountains and highlands of Switzerland and botanical composition of the fodder. *Int. Dairy J.* 12 661–666.

[B16] Conte G. Serra A. Casarosa L. Ciucci F. Cappucci A. Bulleri E. (2019). Effect of linseed supplementation on total longissimus muscle lipid composition and shelf-life of beef from young Maremmana bulls. *Front. Vet. Sci.* 5:326. 10.3389/fvets.2018.00326 30666306 PMC6330289

[B17] Edgar R. C. (2010). Search and clustering orders of magnitude faster than BLAST. *Bioinformatics* 26 2460–2461. 10.1093/bioinformatics/btq461 20709691

[B18] Edgar R. C. (2013). UPARSE: highly accurate OTU sequences from microbial amplicon reads. *Nat. Methods* 10 996–998. 10.1038/nmeth.2604 23955772

[B19] Fox D. G. Sniffen C. J. O’Connor J. D. Russell J. B. Van Soest P. J. (1992). A net carbohydrate and protein system for evaluating cattle diets: III. Cattle requirements and diet adequacy. *J. Anim. Sci.* 70 3578–3596. 10.2527/1992.70113578x 1334063

[B20] Gallo L. De Marchi M. Bittante G. (2014). A survey on feedlot performance of purebred and crossbred european young bulls and heifers managed under intensive conditions in Veneto, northeast Italy. *Ital. J. Anim. Sci.* 13:3285. 10.4081/ijas.2014.3285

[B21] Henderson G. Cox F. Ganesh S. Jonker A. Young W. Collaborators G. R. C. (2015). Rumen microbial community composition varies with diet and host, but a core microbiome is found across a wide geographical range. *Sci. Rep.* 5:14567. 10.1038/srep14567 26449758 PMC4598811

[B22] Holman D. B. Gzyl K. E. (2019). A meta-analysis of the bovine gastrointestinal tract microbiota. *FEMS Microbiol. Ecol.* 95:fiz072. 10.1093/femsec/fiz072 31116403

[B23] Horizon. (2020). *Work Programme 2016 - 2017. Food Security, Sustainable Agriculture and Forestry, Marine and MAritime and Inland Water Research and the Bioeconomy.* Vancouver, BC: Horizon.

[B24] SAS Institute (2008). *User’s Guide: Statistics.* Cary, NC: SAS Institute.

[B25] Jenkins T. C. (1994). Regulation of lipid metabolism in the rumen. *J. Nutr.* 124 1372S–1376S. 8064386 10.1093/jn/124.suppl_8.1372S

[B26] Kabel M. A. Yeoman C. J. Han Y. Dodd D. Abbas C. A. de Bont J. A. M. (2011). Biochemical characterization and relative expression levels of multiple carbohydrate esterases of the xylanolytic rumen bacterium *Prevotella ruminicola* 23 grown on an ester-enriched substrate. *Appl. Environ. Microbiol.* 77 5671–5681. 10.1128/AEM.05321-11 21742923 PMC3165261

[B27] Kaneda T. (1991). Iso- and anteiso-fatty acids in bacteria: biosynthesis, function, and taxonomic significance. *Microbiol. Rev.* 55 288–302. 1886522 10.1128/mr.55.2.288-302.1991 PMC372815

[B28] Kramer J. K. G. Cruz-Hernandez C. Deng Z. Zhou J. Jahreis G. Dugan M. E. R. (2004). Analysis of conjugated linoleic acid and trans 18:1 isomers in synthetic and animal products. *Am. J. Clin. Nutr.* 79 1137S–1145S. 10.1093/ajcn/79.6.1137S 15159247

[B29] Lee M. R. F. Harris L. J. Dewhurst R. J. Merry R. J. Scollan N. D. (2003). The effect of clover silages on long chain fatty acid rumen transformations and digestion in beef steers. *Anim. Sci.* 76 491–501. 10.1017/S1357729800058719

[B30] Li F. Hitch T. C. A. Chen Y. Creevey C. J. Guan L. L. (2019a). Comparative metagenomic and metatranscriptomic analyses reveal the breed effect on the rumen microbiome and its associations with feed efficiency in beef cattle. *Microbiome* 7:6. 10.1186/s40168-019-0618-5 30642389 PMC6332916

[B31] Li F. Li C. Chen Y. Liu J. Zhang C. Irving B. (2019b). Host genetics influence the rumen microbiota and heritable rumen microbial features associate with feed efficiency in cattle. *Microbiome* 7:92. 10.1186/s40168-019-0699-1 31196178 PMC6567441

[B32] Mannelli F. Cappucci A. Pini F. Pastorelli R. Decorosi F. Giovannetti L. (2018). Effect of different types of olive oil pomace dietary supplementation on the rumen microbial community profile in Comisana ewes. *Sci. Rep.* 8:8455. 10.1038/s41598-018-26713-w 29855510 PMC5981327

[B33] Mannelli F. Daghio M. Alves S. P. Bessa R. J. B. Minieri S. Giovannetti L. (2019). Effects of chestnut tannin extract, vescalagin and gallic acid on the dimethyl acetals profile and microbial community composition in rumen liquor: an in vitro study. *Microorganisms* 7: 202. 10.3390/microorganisms7070202 31323805 PMC6680752

[B34] Mao S. Zhang M. Liu J. Zhu W. (2015). Characterising the bacterial microbiota across the gastrointestinal tracts of dairy cattle: membership and potential function. *Sci. Rep.* 5:16116. 10.1038/srep16116 26527325 PMC4630781

[B35] Minato H. Ishibashi S. Hamaoka T. (1988). Cellular fatty acid and sugar composition of representative strains of rumen bacteria. *J. Gen. Appl. Microbiol.* 34 303–319.

[B36] Miyagawa E. (1982). Cellular fatty acid and fatty aldehyde composition of rumen bacteria. *J. Gen. Appl. Microbiol.* 28 389–408.

[B37] Mosley E. E. Powell G. L. Riley M. B. Jenkins T. C. (2002). Microbial biohydrogenation of oleic acid to trans isomers in vitro. *J. Lipid Res.* 43 290–296. 11861671

[B38] Oksanen J. Blanchet F. G. Friendly M. Kindt R. Legendre P. McGlinn D. (2019). *vegan: Community Ecology Package. R package version 2.5-6.*

[B39] Paz H. A. Anderson C. L. Muller M. J. Kononoff P. J. Fernando S. C. (2016). Rumen bacterial community composition in Holstein and Jersey cows is different under same dietary condition and is not affected by sampling method. *Front. Microbiol.* 7:1206. 10.3389/fmicb.2016.01206 27536291 PMC4971436

[B40] Pruesse E. Quast C. Knittel K. Fuchs B. M. Ludwig W. Peplies J. (2007). SILVA: a comprehensive online resource for quality checked and aligned ribosomal RNA sequence data compatible with ARB. *Nucleic Acids Res.* 35 7188–7196. 10.1093/nar/gkm864 17947321 PMC2175337

[B41] R Core Team (2020). *R: A language and Environment for Statistical Computing.* Vienna: R Foundation for Statistical Computing.

[B42] Ramos-Morales E. Arco-Pérez A. Martín-García A. I. Yáñez-Ruiz D. R. Frutos P. Hervás G. (2014). Use of stomach tubing as an alternative to rumen cannulation to study ruminal fermentation and microbiota in sheep and goats. *Anim. Feed Sci. Technol.* 198 57–66. 10.1016/j.anifeedsci.2014.09.016

[B43] Renand G. Havy A. Turin F. (2002). Caractérisation des aptitudes bouchères et qualités de la viande de trois systèmes de production de viande bovine à partir des races rustiques françaises Salers, Aubrac et Gasconne. *INRAE Prod. Anim.* 15 171–183. 10.20870/productions-animales.2002.15.3.3699

[B44] Roehe R. Dewhurst R. J. Duthie C. Rooke J. A. McKain N. Ross D. W. (2016). Bovine host genetic variation influences rumen microbial methane production with best selection criterion for low methane emitting and efficiently feed converting hosts based on metagenomic gene abundance. *PLoS Genet.* 12:e1005846. 10.1371/journal.pgen.1005846 26891056 PMC4758630

[B45] Sargentini C. (2011). “La razza bovina maremmana come produttrice di carne, mediante allevamento in purezza o in incrocio,” in *La razza bovina Maremmana* Editrice Innocenti, (Florence: Quaderni dei Georgofili), 71–84.

[B46] Seshadri R. Leahy S. C. Attwood G. T. Hoong Teh K. Lambie S. C. Cookson A. L. (2018). Cultivation and sequencing of rumen microbiome members from the Hungate1000 Collection. *Nat. Biotechnol.* 36 359–367. 10.1038/nbt.4110 29553575 PMC6118326

[B47] Shabat S. K. B. Sasson G. Doron-Faigenboim A. Durman T. Yaacoby S. Miller M. E. B. (2016). Specific microbiome-dependent mechanisms underlie the energy harvest efficiency of ruminants. *ISME J.* 10 2958–2972. 10.1038/ismej.2016.62 27152936 PMC5148187

[B48] Sofyan A. Uyeno Y. Shinkai T. Hirako M. Kushibiki S. Kanamori H. (2019). Metagenomic profiles of the rumen microbiota during the transition period in low-yield and high-yield dairy cows. *Anim. Sci. J.* 90 1362–1376. 10.1111/asj.13277 31407448

[B49] Stevenson D. M. Weimer P. J. (2007). Dominance of Prevotella and low abundance of classical ruminal bacterial species in the bovine rumen revealed by relative quantification real-time PCR. *Appl. Microbiol. Biotechnol.* 75 165–174. 10.1007/s00253-006-0802-y 17235560

[B50] Tong J. Zhang H. Wang J. Liu Y. Mao S. Xiong B. (2020). Effects of different molecular weights of chitosan on methane production and bacterial community structure in vitro. *J. Integr. Agric.* 19 1644–1655. 10.1016/S2095-3119(20)63174-4

[B51] van Gylswyk N. O. (1995). Succiniclasticum ruminis gen. nov., sp. nov., a ruminal bacterium converting succinate to propionate as the sole energy-yielding mechanism. *Int. J. Syst. Bacteriol.* 45 297–300. 7537062 10.1099/00207713-45-2-297

[B52] Van Soest P. J. Robertson J. B. Lewis B. A. (1991). Methods for dietary fiber, neutral detergent fiber, and nonstarch polysaccharides in relation to animal nutrition. *J. Dairy Sci.* 74 3583–3597. 10.3168/jds.S0022-0302(91)78551-2 1660498

[B53] Vasta V. Daghio M. Cappucci A. Buccioni A. Serra A. Viti C. (2019). Invited review: plant polyphenols and rumen microbiota responsible for fatty acid biohydrogenation, fiber digestion, and methane emission: experimental evidence and methodological approaches. *J. Dairy Sci.* 102 3781–3804. 10.3168/jds.2018-14985 30904293

[B54] Vlaeminck B. Fievez V. Cabrita A. R. J. Fonseca A. J. M. Dewhurst R. J. (2006). Factors affecting odd- and branched-chain fatty acids in milk: a review. *Anim. Feed Sci. Technol.* 131 389–417. 10.1016/j.anifeedsci.2006.06.017

[B55] Zeng B. Li G. Yuan J. Li W. Tang H. Wei H. (2013). Effects of age and strain on the microbiota colonization in an infant human flora-associated mouse model. *Curr. Microbiol.* 67 313–321. 10.1007/s00284-013-0360-3 23604540

[B56] Zhong S. Ding Y. Wang Y. Zhou G. Guo H. Chen Y. (2019). Temperature and humidity index (THI)-induced rumen bacterial community changes in goats. *Appl. Microbiol. Biotechnol.* 103 3193–3203. 10.1007/s00253-019-09673-7 30793235

