# Supplementary material for: Correlation of Breed, Growth Performance, and Rumen Microbiota in Two Rustic Cattle Breeds Reared Under Different Conditions
Source: Front Microbiol. 2021 Apr 29;12:652031. doi: 10.3389/fmicb.2021.652031 (PMC8117017; doi:10.3389/fmicb.2021.652031)
Supplement: Supplementary file 1 [file Data_Sheet_1.DOCX]

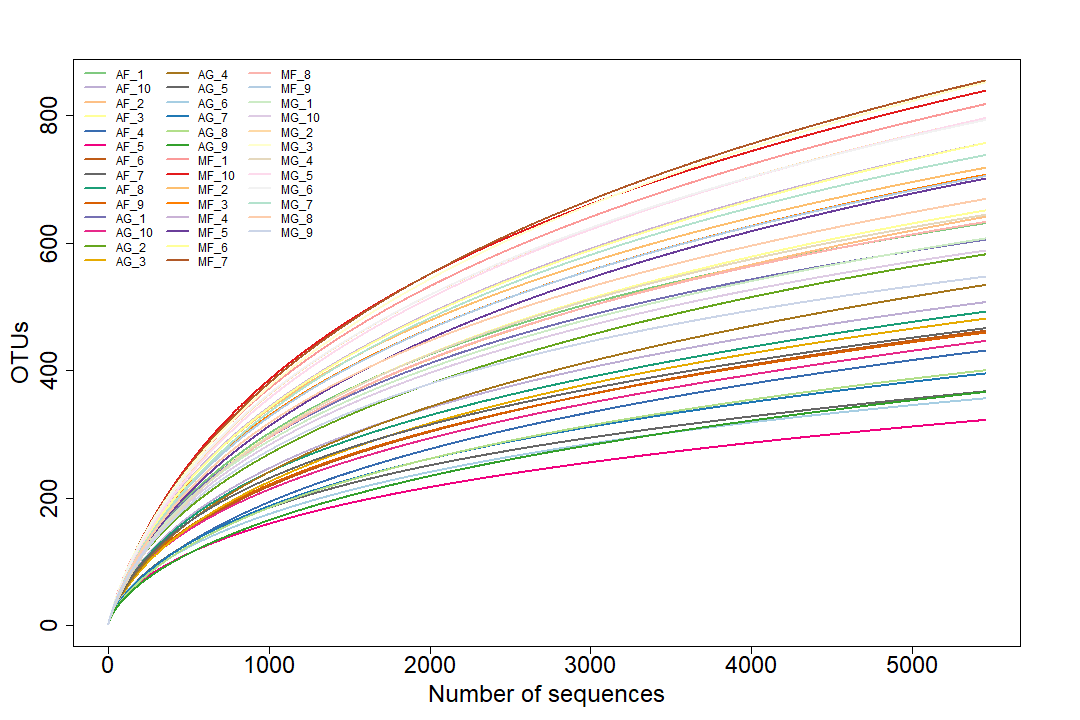


Figure S1 – Rarefaction analysis performed at OTU level on the randomly rarefied dataset (sample size = 5456 sequences). The depth of the sampling was enough to describe the biodiversity within the dataset. Letters and numbers are used to indicate different animals (AF = Aubrac_Feedlot; AG = Aubrac_Grazing; MF = Maremmana_Feedlot; MG = Maremmana_Grazing).
