# Supplementary material for: Correlation of Breed, Growth Performance, and Rumen Microbiota in Two Rustic Cattle Breeds Reared Under Different Conditions
Source: Front Microbiol. 2021 Apr 29;12:652031. doi: 10.3389/fmicb.2021.652031 (PMC8117017; doi:10.3389/fmicb.2021.652031)
Supplement: Supplementary file 2 [file Table_1.DOCX]

Table S1 - Chemical composition and nutritional value of experimental diets (mean ±SD).

| Ingredients (g/100g of DM) | Concentrate | Pasture | Hay |
| --- | --- | --- | --- |
| Flaked corn | 54 | - | - |
| Flaked barley | 8 | - | - |
| Fava bean flour | 19 | - | - |
| Flaked oats | 14 | - | - |
| Wheat Bran | 3 | - | - |
| Calcium carbonate | 2 | - | - |
| Poacee | - | 62.00 | - |
| Fabacee | - | 17.50 | - |
| Other Families | - | 20.50 | - |
|  |  |  |  |
| Chemical composition (% DM) | |  |  |
| ^1^DM (%) | 90.84 ± 3.75 | 20.91 ± 3.5 | 88.97 ± 4.22 |
| ^2^CP (%) | 19.77 ± 2.54 | 16.82 ± 3.77 | 11.33 ± 0.68 |
| ^2^NDF (%) | 22.21 ± 0.32 | 48.17 ± 7.55 | 54.33 ± 0.28 |
| ^2^ADF (%) | 9.02 ± 1.397 | 29.175 ± 3.22 | 39.09 ± 5.87 |
| ^2^ADL (%) | 1.99 ± 0.98 | 8.21 ± 2.18 | 8.33 ± 3.87 |
| ^2^EE (%) | 2.08 ± 0.21 | 1.86 ± 0.53 | 1.35 ± 0.54 |
| ^2^Ash (%) | 14.85 ± 0.72 | 8.075 ± 0.87 | 8.41 ± 1.12 |
| NE^3^ | 2.1 + 0.05 | 1.3 + 0.04 | 1.2 + 0.06 |
| Fatty acids (g/100g of total fatty acids) | |  |  |
| C16:0 | 19.79 ± 2.73 | 15.8 ± 2.04 | 19.56 ± 2.73 |
| C18:0 | 1.64 ± 0.23 | 2.52 ± 0.33 | 3.24 ± 0.45 |
| C18:1 *cis*9 | 14.37 ± 1.98 | 4.96 ± 0.97 | 3.29 ± 0.45 |
| C18:2 *n*-6 | 49.43 ± 6.82 | 19.48 ± 2.51 | 6.65 ± 0.92 |
| C18:3 *n*-3 | 3.42 ± 0.47 | 47.65 ± 6.07 | 7.18 ± 0.99 |

DM – dry matter; CP – crude protein; NDF – neutral detergent fiber; ADF – acid detergent fiber; ADL – acid detergent lignin; NE – net energy;. SD – standard deviation; ^1^Expressed as g/100g of fresh weight.^2^Expressed as g/100g of DM.^3^Expressed as Mcal/kg of DM ADF – acid detergent fiber.
