# Supplementary material for: Correlation of Breed, Growth Performance, and Rumen Microbiota in Two Rustic Cattle Breeds Reared Under Different Conditions
Source: Front Microbiol. 2021 Apr 29;12:652031. doi: 10.3389/fmicb.2021.652031 (PMC8117017; doi:10.3389/fmicb.2021.652031)
Supplement: Supplementary file 3 [file Table_2.DOCX]

Table S2 – Composition of bacterial communities at family level. The relative abundance calculated for the Aubrac breed and for the Maremmana breed in the two rearing systems. Only the families with an average relative abundance of 1%, or higher, in at least one group (i.e., Aubrac grazing, Aubrac feedlot, Maremmana grazing, Maremmana feedlot) are reported.

|  | AU | | MA | |  | P value | | |
| --- | --- | --- | --- | --- | --- | --- | --- | --- |
| Family | Grazing (%) | Feedlot (%) | Grazing (%) | Feedlot (%) | SE | B | R | BxR |
| *Bacteroidales* RF16 group | 2.79 | 2.99 | 1.95 | 1.59 | 0.34 | 0.208 | 0.140 | 0.289 |
| F082 | 4.31 ^b^ | 3.85 ^b^ | 8.78 ^a^ | 10.69 ^a^ | 0.61 | <0.001 | 0.776 | <0.001 |
| *Muribaculaceae* | 6.35 | 8.13 | 5.56 | 9.00 | 0.85 | 0.245 | 0.070 | 0.103 |
| p-251-o5 | 0.01 ^b^ | 0.02 ^b^ | 2.76 ^a^ | 0.30 ^a^ | 0.42 | <0.001 | 0.543 | <0.001 |
| *Prevotellaceae* | 39.83 ^a^ | 38.36 ^a^ | 18.99 ^b^ | 15.51 ^b^ | 2.17 | <0.001 | 0.291 | <0.001 |
| *Rikenellaceae* | 6.11 ^c^ | 6.72 ^bc^ | 12.46 ^a^ | 9.84 ^ab^ | 0.58 | <0.001 | 0.552 | <0.001 |
| *Fibrobacteraceae* | 4.67 ^a^ | 2.97 ^a^ | 0.72 ^b^ | 0.71 ^b^ | 0.51 | <0.001 | 0.123 | <0.001 |
| *Acidaminococcaceae* | 3.71 ^a^ | 4.25 ^a^ | 0.82 ^b^ | 0.53 ^b^ | 0.40 | <0.001 | 0.839 | <0.001 |
| *Christensenellaceae* | 2.01 ^b^ | 1.68 ^b^ | 6.98 ^a^ | 7.72 ^a^ | 0.51 | <0.001 | 0.695 | <0.001 |
| Family XIII | 0.61 ^b^ | 0.66 ^b^ | 1.71 ^a^ | 2.12 ^a^ | 0.12 | <0.001 | 0.323 | <0.001 |
| *Lachnospiraceae* | 9.75 | 8.04 | 9.23 | 9.74 | 0.44 | 0.344 | 0.766 | 0.629 |
| *Ruminococcaceae* | 10.19 ^b^ | 11.78 ^b^ | 21.00 ^a^ | 22.79 ^a^ | 1.04 | <0.001 | 0.499 | <0.001 |
| *Veillonellaceae* | 1.09 ^a^ | 1.32 ^a^ | 0.36 ^b^ | 0.26 ^b^ | 0.13 | <0.001 | 0.839 | <0.001 |
| *Saccharimonadaceae* | 1.06 ^b^ | 1.05 ^b^ | 2.19 ^ab^ | 3.84 ^a^ | 0.24 | <0.001 | 0.204 | <0.001 |
| *Succinivibrionaceae* | 1.23 ^a^ | 0.94 ^a^ | 0.01 ^b^ | 0.04 ^b^ | 0.29 | <0.001 | 0.531 | <0.001 |
| *Spirochaetaceae* | 2.18 | 2.46 | 0.85 | 1.00 | 0.37 | 0.113 | 0.839 | 0.280 |
| Other Families | 1.80 | 2.17 | 3.88 | 2.61 | 0.21 | N.A. | N.A. | N.A. |
| Unclassified | 2.29 | 2.60 | 1.77 | 1.72 | 0.16 | 0.045 | 0.490 | 0.144 |

AU (Aubrac), MA (Maremmana), SE (Standard Error), B (breed), R (rearing system), N.A. (not available). a, b, c is the probability of significant effect due to interaction BxR; means within a row with different letters differ (p < 0.05).
