# Supplementary material for: Correlation of Breed, Growth Performance, and Rumen Microbiota in Two Rustic Cattle Breeds Reared Under Different Conditions
Source: Front Microbiol. 2021 Apr 29;12:652031. doi: 10.3389/fmicb.2021.652031 (PMC8117017; doi:10.3389/fmicb.2021.652031)
Supplement: Supplementary file 4 [file Table_3.DOCX]

Table S3 - Composition of bacterial communities at genus level. The relative abundance calculated for the Aubrac breed and for the Maremmana breed in the two rearing systems. Only the genera with an average relative abundance of 1%, or higher, in at least one group (i.e., Aubrac grazing, Aubrac feedlot, Maremmana grazing, Maremmana feedlot) are reported.

|  | AU | | MA | |  | P value | | |
| --- | --- | --- | --- | --- | --- | --- | --- | --- |
| Genus | Grazing (%) | Feedlot (%) | Grazing (%) | Feedlot (%) | SE | B | R | BxR |
| *Prevotella* 1 | 28.22 ^a^ | 26.69 ^a^ | 14.09 ^b^ | 10.83 ^b^ | 1.80 | <0.001 | 0.304 | <0.001 |
| *Prevotella* 7 | 5.39 ^a^ | 3.94 ^a^ | N.D. ^b^ | <0.01 ^b^ | 0.85 | <0.001 | 0.712 | <0.001 |
| *Prevotellaceae* UCG-001 | 0.87 | 0.84 | 1.11 | 1.24 | 0.08 | 0.064 | 0.882 | 0.307 |
| *Prevotellaceae* UCG-003 | 1.92 | 1.90 | 2.21 | 1.25 | 0.18 | 0.665 | 0.148 | 0.238 |
| *Rikenellaceae* RC9 gut group | 6.10 ^c^ | 6.69 ^bc^ | 12.42 ^a^ | 9.78 ^ab^ | 0.58 | <0.001 | 0.543 | <0.001 |
| *Fibrobacter* | 4.67 ^a^ | 2.97 ^a^ | 0.70 ^b^ | 0.71 ^b^ | 0.51 | <0.001 | 0.137 | <0.001 |
| *Acetitomaculum* | 0.92 | 0.94 | 1.02 | 1.22 | 0.13 | 0.034 | 0.409 | 0.155 |
| *Christensenellaceae* R-7 group | 1.99 ^b^ | 1.66 ^b^ | 6.81 ^a^ | 7.64 ^a^ | 0.50 | <0.001 | 0.655 | <0.001 |
| *Lachnospiraceae* NK3A20 group | 0.66 ^b^ | 0.78 ^b^ | 1.17 ^ab^ | 2.00 ^a^ | 0.14 | <0.001 | 0.229 | <0.001 |
| *Oribacterium* | 1.40 ^a^ | 0.84 ^a^ | 0.07 ^b^ | 0.04 ^b^ | 0.18 | <0.001 | 0.881 | <0.001 |
| *Ruminococcaceae* NK4A214 group | 2.35 ^b^ | 2.13 ^b^ | 7.61 ^a^ | 8.06 ^a^ | 0.52 | <0.001 | 0.892 | <0.001 |
| *Ruminococcaceae* UCG-010 | 0.50 ^b^ | 0.50 ^b^ | 1.23 ^a^ | 1.09 ^a^ | 0.09 | <0.001 | 0.935 | 0.002 |
| *Ruminococcaceae* UCG-014 | 1.44 | 2.02 | 2.11 | 2.28 | 0.23 | 0.130 | 0.086 | 0.122 |
| *Ruminococcus* 1 | 1.79 ^a^ | 1.25 ^ab^ | 0.49 ^c^ | 0.54 ^bc^ | 0.14 | <0.001 | 0.735 | <0.001 |
| *Ruminococcus* 2 | 0.21 ^b^ | 0.40 ^b^ | 2.69 ^a^ | 2.62 ^a^ | 0.26 | <0.001 | 0.645 | <0.001 |
| *Saccharofermentans* | 1.34 | 1.85 | 2.13 | 2.38 | 0.19 | 0.037 | 0.137 | 0.081 |
| *Succiniclasticum* | 3.41 ^a^ | 4.00 ^a^ | 0.81 ^b^ | 0.53 ^b^ | 0.39 | <0.001 | 0.860 | <0.001 |
| *Candidatus Saccharimonas* | 1.06 ^b^ | 1.05 ^b^ | 2.19 ^ab^ | 3.84 ^a^ | 0.24 | <0.001 | 0.204 | <0.001 |
| *Succinivibrionaceae* UCG-002 | 1.05 | 0.02 | <0.01 | <0.01 | 0.25 | 0.444 | 0.896 | 0.662 |
| *Treponema* 2 | 2.16 | 2.41 | 0.75 | 0.88 | 0.37 | 0.051 | 0.871 | 0.181 |
| Other Genera | 7.87 | 10.34 | 11.09 | 10.41 | 0.39 | N.A. | N.A. | N.A. |
| Unclassified | 24.70 ^b^ | 26.78 ^b^ | 29.30 ^ab^ | 32.65 ^a^ | 1.09 | 0.009 | 0.164 | 0.020 |

AU (Aubrac), MA (Maremmana), SE (Standard Error), B (breed), R (rearing system), N.D. (not detected), N.A. (not available). a, b, c, is the probability of significant effect due to interaction BxR; means within a row with different letters differ (p < 0.05).
