# Supplementary material for: Correlation of Breed, Growth Performance, and Rumen Microbiota in Two Rustic Cattle Breeds Reared Under Different Conditions
Source: Front Microbiol. 2021 Apr 29;12:652031. doi: 10.3389/fmicb.2021.652031 (PMC8117017; doi:10.3389/fmicb.2021.652031)
Supplement: Supplementary file 5 [file Table_4.docx]

Table S4 – *In vivo* performance calculated for the Aubrac (AU) breed and for the Maremmana (MA) breed in the two rearing systems.

|  | AU | | MA | | SE | p-value | | |
| --- | --- | --- | --- | --- | --- | --- | --- | --- |
|  | Feedlot | Grazing | Feedlot | Grazing |  | B | R | BxR |
| Initial weight (Kg) | 440.9 | 455.1 | 331.7 | 333.3 | 10.80 | <0.001 | 0.404 | 0.327 |
| Final weight (Kg) | 628.2 | 623.3 | 524.2 | 548.9 | 15.14 | <0.001 | 0.544 | 0.329 |
| ADG (Kg) | 0.90 | 0.99 | 0.89 | 0.84 | 0.07 | 0.049 | 0.836 | 0.140 |

ADG (average daily weight gain), AU (Aubrac), MA(Maremmana), SE (Standard Error), B (breed) and R (rearing system).
